# Soldering Crown and Bridge Work

**Published:** 1906-05

**Authors:** L. A. Neill

**Affiliations:** New Decatur.


### SOLDERING CROWN AND BRIDGE WORK.

By L. A. Neill, D. D. S., New Decatur.

Alabama Dental Association.

The ideas which I desire to set forth in this paper have been of such assistance to me that I wish to present them to this honorable body. It is not my intention to give methods of construction, but to set forth ways by which crowns and bridges may be soldered with ease without melting bands or backing and without cracking porcelain facings.

In soldering bands of crowns for bridge work, I lap the joint of the band and hammer the margin thin on the horn of the small anvil, using 22K gold for the band and 22K solder for soldering. I use for the cap of crowns a piece of thirty-six gauge platinum that will just cover the top of the band, on which I place little balls of 22K gold (which I make of scraps) at each point where I wish a cusp, flowing them with a blowpipe until they spread over the surface of the platinum, stopping in time to have a well-defined cusp. I solder this on with 22K solder. In this way we have a crown that subsequent soldering will not change.

When the work is set up ready for investment, the model should be trimmed away, leaving just enough to hold the crowns and teeth in place. Invest with equal parts of marble dust and plaster. When well hardened, all wax may be removed by slowly pouring boiling water on it until every particle of wax is washed away. All surplus investment should be cut away, so as to leave as much of the metal exposed to the flame as possible.

Never attempt to solder a bridge by forcing the flame down into hole, for it is one of the most difficult things to do. The more play and freedom the flame has, the better will be the results. ,

Care should be taken to have all portions of the porcelain covered, also to fill all crevices between the porcelain even with the surface of the backing, wetting with a solution of borax all surfaces to be soldered.

Solder cut into convenient-sized pieces should be mixed thoroughly with a borax solution and placed near the heater to dry. A wet or new investment is less liable to cause teeth to check than a dry one, so I prefer soldering as soon as the work is invested.

When all these points have been carefully observed, the case should be placed to heat upon a gas stove or burner, being sure that the flame does not strike the teeth or backing directly. It must first heat the investment, letting that in turn heat the porcelain and metal. When the work is heated until the metal exposed is red, the case may be moved to a soldering block; or, if preferred, it may be soldered on the burner where it was heated. It is best to commence at one end, laying on a piece or two of solder, being sure that the first pieces of solder flow before putting on others, for here begins the good or poor soldered case.

Solder should never be forced with a small flame of the blowpipe, as a large one will do the work very much better without burning the crowns or backing When the solder is once flowed to a band, it should be left there. If not satisfactory, another piece should be used, even if it has to be ground off later.

Solder once flowed will not move as easily the second time, and, if attempted, is liable to take a part of the band with it. Soldering should always be finished at one point before leaving it, gradually shifting the position of the case so as to work toward the other end. Never start at both ends and finish in the middle.

A little instrument made of German silver wire, about size 14, with platinum wire two inches long for a point, is very useful to draw the solder upon the crowns when extra strength or thickness is needed, also to keep the borax on the surface, thereby avoiding pit holes.

After the soldering is completed, a uniform cooling is essential. The best and safest way is to bury immediately in a can of plaster or marble dust, leaving it there to cool.

